# Trib1 Contributes to Recovery From Ischemia/Reperfusion-Induced Acute Kidney Injury by Regulating the Polarization of Renal Macrophages

**DOI:** 10.3389/fimmu.2020.00473

**Published:** 2020-03-20

**Authors:** Xiangcheng Xie, Xiu Yang, Junxia Wu, Jilin Ma, Wei Wei, Xiao Fei, Ming Wang

**Affiliations:** ^1^Department of Nephrology, Affiliated Hangzhou First People's Hospital, Zhejiang University School of Medicine, Hangzhou, China; ^2^Department of Nephrology, The Second Affiliated Hospital, School of Medicine, Zhejiang University, Hangzhou, China; ^3^Division of Nephrology, Zhejiang Traditional Chinese Medicine and Western Medicine Hospital, Hangzhou, China

**Keywords:** acute kidney injury, adaptive self-repair, Trib1, tubular epithelial cell, cell proliferation, macrophage polarization

## Abstract

Increasing evidence suggests that macrophage polarization is involved in the recovery from ischemia-reperfusion (I/R)-induced acute kidney injury (AKI), implying that the regulation of macrophage polarization homeostasis might mediate AKI recovery. Trib1 is a key regulator of macrophage differentiation, but its role in AKI remains unclear. Here, we aimed to investigate the role of Trib1 and its link with the macrophage phenotype in the process of adaptive recovery from I/R-induced renal injury. Lentiviral vector-mediated RNA interference (RNAi) was used to knock down Trib1 expression *in vitro* and *in vivo*, and a mouse model of moderate AKI was established by the induction of I/R injury. Renal function measurements and inflammatory factors were determined by the corresponding kits. Histomorphology was assessed by hematoxylin-eosin, Masson and PAS staining. Western blot and flow cytometry were employed for the analysis of signal transduction, cell apoptosis and macrophage phenotypes. Trib1 knockdown inhibited cell viability of tubular epithelial cells (TECs) by inhibiting proliferation and enhancing apoptosis *in vitro*. I/R-induced AKI significantly impaired renal function in mice via increasing the levels of BUN, Scr, NGAL and renal tubular damage, leading to renal fibrosis from days 1 to 3. Through the adaptive self-repair mechanism, renal dysfunction recovered over time and returned to almost normal levels on day 28 after I/R intervention. However, Trib1 depletion worsened renal damage on day 3 and blunted the adaptive repair process of the renal tissue. Mechanistically, Trib1 inhibition suppressed renal tubular cell proliferation under adaptive self-repair conditions by affecting the expression of the proliferation-related proteins cyclin D1, cyclin B, p21, and p27, the apoptosis-related proteins Bcl-2 and Bax, and the fibrosis-related proteins collagen I and III. Furthermore, the M1/M2 macrophage ratio increased in the first 3 days and decreased from day 7 to day 28, consistent with changes in the expression of inflammatory factors, including TNFα, IL-6, IL-12, IL-10, and IL-13. Trib1 inhibition blocked macrophage polarization during adaptive recovery from I/R-induced moderate AKI. Our results show that Trib1 plays a role in kidney recovery and regeneration via the regulation of renal tubular cell proliferation by affecting macrophage polarization. Thus, Trib1 might be a viable therapeutic target to improve renal adaptive repair following I/R injury.

## Introduction

Acute kidney injury (AKI) is one of the most common serious complications, with increased risks of short- and long-term mortality in hospitalized patients (1). Approximately 20~50% of patients who experience maladaptive repair of AKI may continue to develop chronic kidney disease (CKD), which is associated with an increased risk of end-stage renal disease (ESRD) (2). Renal ischemia-reperfusion injury (IRI) facilitates the development of AKI and is accompanied by significantly decreased TECs numbers (3). The regenerative ability of TECs is a determining element for the adaptive repair of progressive AKI (4). Therefore, complete repair after AKI by improving the activity of TECs is a useful therapeutic approach for preventing or treating AKI.

The repair process following IRI mainly comprises two major events: the resolution of local inflammation and the regeneration of TECs (5), and tissue inflammation is crucial for the pathogenesis of I/R-induced renal injury (6). Infiltrating cells, especially macrophages, act as an important determinant in the initiation and propagation of AKI. Macrophages participate in not only the initial injury after I/R by secreting inflammatory cytokines and inducing TEC apoptosis but also in tubular repair after I/R by regulating the immune response to inflammation (7). Macrophages are functionally classified into two categories: M1 (inflammatory) and M2 (anti-inflammatory). M1 macrophages play a pathogenic role in boosting inflammatory and renal injuries, whereas M2 macrophages exhibit anti-inflammatory and wound-healing effects (8). The increased levels of inflammatory factors, including IL-4 and IL-13, inhibit recovery from I/R-induced AKI, which is associated with increased M1 and decreased M2 marker expression (9). Evidence demonstrates that blockage of the initial macrophage influx abates I/R-mediated renal injury (10). Macrophage depletion at early time points leads to the remission of renal damage, whereas macrophage removal at later time points results in impaired recovery, implying that macrophage polarization plays a critical role during the I/R-induced AKI recovery process (11). Additionally, recent studies have demonstrated crosstalk between the tubular epithelium and interstitial cells, and TECs interact with macrophages during both AKI repair/regeneration and disease progression (12, 13). In contrast, injured TECs can cause the activation of M1 macrophages in renal injury (14). Hence, the cellular homeostasis of macrophage polarization might contribute to the promotion of TEC proliferation and renal recovery after I/R.

Tribbles homolog 1 (Trib1) is an adaptor protein involved in the protein degradation of immune-related transcription factors and is critical for the differentiation of macrophages. Trib1 depletion increases the neutrophil population in the spleen but decreases the M2-like macrophage population (15). *Trib1* deficiency in mice causes severe liver fibrosis via regulation of the macrophage population and differentiation in the liver (16). Trib1 is a key switch for adipose tissue maintenance and metabolic disorder inhibition that functions by modulating the polarization of tissue-resident M2-like macrophages (17). In addition, as a biomarker of chronic antibody-mediated rejection, Trib1 expression is upregulated in a rodent model of chronic cardiac vasculopathy and may be a useful biomarker in other types of solid-organ transplantation, including renal transplantion (18). Given the role of Trib1 in renal injury and macrophage differentiation, we hypothesized that Trib1 may be involved in the recovery of AKI through crosstalk between macrophage phenotypes.

## Materials and Methods

### Cell Culture, Transfection, and Treatment

Mouse renal tubular epithelial cell line TCMK-1 was purchased from the Shanghai Institute for Biological Sciences, Chinese Academy of Sciences. The cells were incubated in complete DMEM medium supplemented with 10% FBS at 37°C with 5% CO_2_. Prior to viral infection, cells were seeded in 6-wells plate at the density of 0.5 x 10^6^ cells/well allowing to grown to 70–80% confluence. For the construction of lentivirus packaging Trib1 shRNA, control scrambled shRNA and small hairpin RNAs (shRNAs) specifically targeting Trib1 were synthesized and inserted into the pLKO.1-EGFP lentiviral vector. For lentiviral particles production, the expression plasmids (Trib1-pLKO.1-EGFP and negative control plasmid) and packaging vectors pCMV-VSV-G and pCMV-dR8.2 (Addgene, Cambridge, MA, US) were co-transfected into 293T cells using Lipofectamin^TM^ 2000 (Invitrogen, US). After transfection for 48 h, the supernatant containing lentiviral particles were collected and centrifuged at 4,000 × g for 5 min at 4°C, and then filtered with 0.45-μm cellulose acetate filters to eliminate cell debris. The collected lentiviral particles were purified and concentrated utilizing PEG-8000 according to the manufacturer's instructions. Then, viral stock containing Trib1-shRNA at a multiplicity of infection (MOI) of 100 were added to the cells. At end of 48 h of the incubation, infection efficiency was determined by western blotting. For the *in vitro* IRI model by enzymatic method, Briefly, GOX/CAT system consisting of glucose oxidase (GOX) and catalase (CAT) and 2-deoxyglucose (a non-metabolizable isomer of L-glucose) were used. Firstly, glucose oxidase (3 mM/s) and catalase (998 s-1) were diluted at a constant 10:1 ratio in cell culture medium. The induction of ischemia was achieved by incubating cells in DMEM without glucose and 2-deoxyglucose but supplemented with GOX/CAT for 1 h and the *in vitro* reperfusion was achieved by incubating cells in glucose-replete complete growth medium for 3 h. For the recovery assay, cells were preincubated with 1 mM NaSH (positive control) and NaCl (negative control), respectively, for 1 h before the induction of 1 h ischemia and 3 h reperfusion.

### CCK8 Assay

Transfected TCMK-1 cells with NC-shRNA or Trib1-shRNA were seeded in 96-well plate at the density of 2 × 10^3^ cells/well. After incubation overnight, cells viability was detected by CCK-8 kit according to the manufacture's instruction (CA1210, Solarbio, China) after incubation for 0 and 24 h. The absorbance was measured at the wavelength of 450 nm by a Multiscan plate reader (SynergyTM H1, BioTek, USA).

### Mouse Model and Treatments

Male C57BL/6 mice were purchased from Hubei Provincial Laboratory Animal Public Service Center and housed at an ambient temperature of 20°C and a relative air humidity of 60% with a 12 h light rhythm cycle (7:00 a.m. to 7:00 p.m.). For the Trib1-deficient mice, a green fluorescent protein (GFP)-labeled lentivirus targeting Trib1 was first constructed, harvested and purified after transfection into 293T cells. Then, 4-week-old mice were injected with 0.2 ml of a lentivirus packaging plasmid at 1 × 10^8^ PFU/ml twice a week for 4 consecutive weeks. At 8 weeks of age, the mice were randomly divided into five groups: sham, moderate IRI, Trib1 depletion (Trib1 shRNA), moderate IRI plus Trib1-NC shRNA, and moderate IRI plus Trib1 shRNA. After anesthetization, ischemia was induced in the left kidney by the retroperitoneal approach with a non-traumatic microaneurysm clip for 30 min at 37°C (unilateral IRI). After the clamps were removed and reperfusion of the left kidney was confirmed, the wounds were sutured. Mice in the sham group underwent the same procedure without clamping. All mice had free access to food and water. On days 1, 3, 7, 14, and 28 after surgery, the mice were sacrificed, and renal tissue and blood samples were collected for subsequent analysis. To maintain the knockdown efficiency of Trib1, mice were injected with lentivirus every 5 days during the model period. The animal experiments were approved by the Ethics Committee of Hangzhou First People's Hospital. The investigation was performed in accordance with the Guide for the Care and Use of Laboratory Animals published by the National Academies Press (8th Edition, 2010).

### Scr, BUN, and NGAL Measurements

Briefly, blood plasma was collected by the abdominal aortic method and incubated for 2 h at room temperature. After centrifugation at 3,500 rpm for 5 min, serum creatinine (Scr), blood urea nitrogen (BUN) and neutrophil gelatinase-associated lipocalin (NGAL) levels were measured by using the corresponding detection kits (Scr: C011-1, Jiangcheng Bio, Nanjing; BUN: C013-2, Jiangcheng, Nanjing; NGAL: ml002141, Mibio, Shanghai) according to the protocols specified by the manufacturer.

### Histomorphological Staining

Renal tissues were fixed in methyl Carnoy's solution, and paraffin-embedded renal sections (5-μm thick) from the above five groups were cut and stained with hematoxylin-eosin (HE) and Masson's trichrome and periodic acid-Schiff (PAS) as previously described (19). HE and PAS staining was used to evaluate the injury and inflammatory cell infiltration in renal tissues from the different groups. Masson's trichrome staining was employed to determine the degree of renal fibrosis. Pathological histology was observed using a biological microscope. The acute tubular necrosis (ATN) score was graded by proximal tubule dilation, brush border damage, proteinacenous casts, interstitial widening, and necrosis which was scored as follows: 0, none necrosis; 1, <11% necrosis; 2, 11–25% necrosis; 3, 26–45% necrosis; 4, 46–75% necrosis; 5, >75% necrosis. The specimens were evaluated in a blinded manner to the mouse strain. The tubulointerstitial damage degree can be divided into the quality grade 0 (normal), grade 1 (damage area <10%), grade 2 (damage area: 10%~30%), grade 3 (damage area: 30%~50%), and grade 4 (damage area>50%). The damage area was performed using double blind experiment and assessed by two pathologists through observing five horizons in each section (*n* = 5 per group). And the assessment result was verified by another two pathologists.

### Western Blot Analysis

Total protein was extracted from the renal tissues and renal tubular epithelial cells using RIPA lysis buffer (Beyotime, China), and the concentration was estimated using a BCA Protein Assay kit (Beyotime, China). Equal amounts of protein (40 μg) were separated by sodium dodecyl sulfate-polyacrylamide gel electrophoresis (SDS-PAGE) and then transferred to polyvinylidene difluoride membranes (PVDF). After blocking with 5% skim milk, the membranes were incubated with primary antibodies against cyclin D1 (ab40754, Abcam, China), cyclin B (ab2096, Abcam, China), p21 (ab188224, Abcam, China), Bcl-2 (ab182858, Abcam, China), Bax (ab182733, Abcam, China), collagen I (ab34710, Abcam, China), collagen III (ab7778, Abcam, China) and Trib1 (ab137717, Abcam, China). GAPDH (ab8245, Abcam, China) served as the internal control. After three wash cycles, the membranes were incubated with horseradish peroxidase-conjugated goat anti-mouse or goat anti-rabbit antibodies for 2 h at room temperature. Then, protein expression was visualized with an enhanced chemiluminescence reporter system detection kit. The band intensities were examined by TINA image software (Raytest, Straubenhardt, Germany) and normalized to that of GAPDH.

### ELISA

The concentrations of TNF-α (ml002095, Mibio, Shanghai), IL-6 (ml063159, Mibio, Shanghai), IL-12 (ml037868, Mibio, Shanghai), IL-4 (ml063156, Mibio, Shanghai), IL-10 (ml037873, Mibio, Shanghai) and IL-13 (ml063123, Mibio, Shanghai) in plasma were determined by enzyme-linked immunosorbent assay kits according to the manufacturer's protocols. The OD values of different indexes were measured using a Victor 3 multilabel plate reader (Perkin Elmer).

### qRT-PCR

Total RNA was extracted using TRIzol Reagent One-Step, and a reverse transcription reaction was conducted according to the Fermentas Corporation M-MLV protocol. The primers were designed with Primer Premier 5.0 software (Premier Biosoft International, Palo Alto, CA, USA), and the iNOS, Ccl3, Arg1, and Igf2r primer sequences are shown in Table 1. Amplification reactions were performed in a 50 μl reaction volume using the SYBR Green I method. The reaction conditions were as follows: predenaturation at 95°C for 2 min; 40 cycles of denaturation at 95°C for 15 s, annealing at 60°C for 30 s and extension at 72°C for 30 s; and a final hold at 4°C. The relative mRNA expression levels were calculated using the 2^−ΔΔCT^ method with GAPDH as the normalization control.

**Table 1:** Primer sequences for related genes.

**Genes**	**Sequences**	
iNOS	Forward: 5′-3′	AATCTTGGAGCGAGTTGTGG
	Reverse: 5′-3′	CAGGAAGTAGGTGAGGGCTTG
Ccl3	Forward: 5′-3′	CCAGCCAGGTGTCATTTTCC
	Reverse: 5′-3′	AGGCATTCAGTTCCAGGTCA
Arg1	Forward: 5′-3′	GGAAGACAGCAGAGGAGGTG
	Reverse: 5′-3′	TATGGTTACCCTCCCGTTGA
Igf2r	Forward: 5′-3′	ACTGATGGTGATGACTGTGGC
	Reverse: 5′-3′	TTGCAGCTCTTAGCACTGGAG
GAPDH	Forward: 5′-3′	AAGACGGGCGGAGAGAAACC
	Reverse: 5′-3′	CGTTGACTCCGACCTTCACC

### Flow Cytometry

To quantify the polarization of renal macrophages in mouse kidneys from different groups, renal tissues were minced into 1 mm^3^ fragments and digested in RMPI 1640 buffer containing 2 mg/ml collagenase type D and 100 U/ml DNase I for 1 h at 37°C. The digested kidneys were then sequentially passed through a 70-μm mesh, yielding single-cell suspensions. Red blood cells in the resulting renal cell suspension were lysed with red blood cell lysis buffer (Sigma, US). Renal macrophages were centrifuged and resuspended in FACS buffer on ice. After incubation with 2.5 μg/ml Fc-blocking solution, the cells were resuspended in FACS buffer. Then, 10^6^ cells were stained with the following fluorochrome-labeled antibodies: F4/80 (eBiosciences)-PE, iNOS-FITC, and CD206-FITC. After immunostaining, the cells were analyzed immediately on a FACS Canto II cytometer with DIVA software (Becton Dickinson), and the data were analyzed using Cyflogic V.1.2.1 software. For apoptosis assay, cells exposed with different reagents were digested washed with PBS for twice. Then cells were resuspended using 500 μL annexin V binding buffer and stained with Annexin V-APC Apoptosis Detection Kit according to the manufacturer's protocols (KeyGEN, Nanjing, Jiangsu, China). Then the proportion of apoptotic cells were detected by flow cytometry and analyzed using ModFit LT software (Becton Dickinson, Mountain View, CA, USA).

### Statistical Analysis

All data are expressed as the mean ± standard deviation (SD). One-way analysis of variance (ANOVA) followed by a *post hoc* Student-Newman-Keuls multiple comparisons test was used to evaluate the differences between groups using SPSS software (V21.0, SPSS, Inc., IL, USA). If data were not normally distributed, comparisons between the experimental and control groups were performed using Kruskal-Wallis one-way analysis followed by Dunn's test. Statistical significance was defined as *p* < 0.05.

## Results

### Trib1 Inhibition Impedes Cell Viability of Renal Tubular Epithelial Cell by Depressing Proliferation and Enhancing Apoptosis

**Figure 1:**
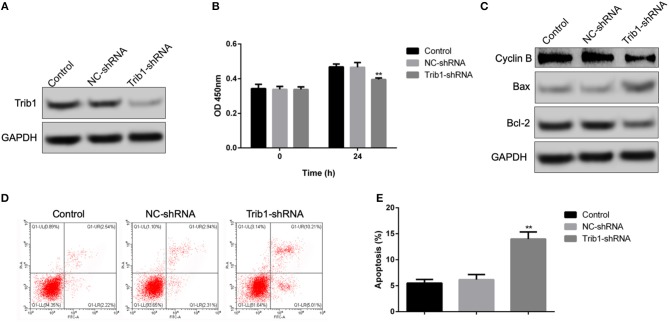
Effect of Trib1 knockdown on cell proliferation and apoptosis of renal tubular epithelial cells *in vitro*. **(A)** The knockdown efficiency of lentivirus-mediated transfection of Trib1-shRNA as analyzed by western blotting in renal tubular epithelial cells. **(B)** Cell viability as measured by CCK8 assay in renal tubular epithelial cells transfected with NC-Trib1 and Trib1-shRNA. **(C)** Protein expression of BAX, Bcl-2 and Cyclin B as detected by western blotting. **(D)** Cell apoptosis induced by Trib1 knockdown as determined by flow cytometry. **(E)** Quantitative analysis of apoptotic cell proportion in panel d. * indicates the NC-Trib1 group vs the Trib1-shRNA group. *p < 0.05; **p < 0.01.

During the process of renal self-repair after AKI, the cell viability of renal tubular epithelial cells plays a decisive role. Thus, we first explored the role of Trib1 on cell proliferation and apoptosis of renal tubular epithelial cells using a lentivirus-mediated transfection of Trib1-shRNA. As shown in Supplementary Figure 1, after transfection with Trib1-shRNA, the expression of Trib1 in renal tubular epithelial cells was robustly depressed (Figure 1A). Combined with CCK8 assay, we observed that Trib1 removal significantly inhibited cell viability of renal tubular epithelial cells (Figure 1B). Additionally, Trib1 depletion also caused the increase of Bax, the decrease of Bcl-2 and Cyclin B compared to control or NC-shRNA groups (Figure 1C). Thus, Trib1 might act as a positive regulator in cell viability of renal tubular epithelial cells by depressing proliferation and activating apoptosis *in vitro*.

### Trib1 Knockdown Impairs the Adaptive Repair of Renal Dysfunction Induced by Acute Kidney Injury

**Figure 2:**
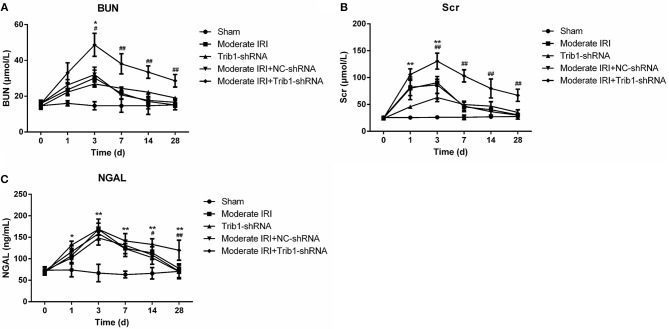
Serum index measurements of renal function in mice. Mice were exposed to I/R, Trib1 shRNA, and I/R plus Trib1 shRNA. Then, the mice were randomly divided into five groups: sham, Trib1 shRNA, moderate IRI, moderate IRI+Trib1 NC shRNA and moderate IRI+Trib1 shRNA. The serum indexes, including **(A)** Scr, **(B)** BUN and **(C)** NGAL, were measured by the corresponding kit at each time point. * Indicates the IRI+Trib1 shRNA group vs. the sham group; # indicates the IRI+Trib1 shRNA group vs. the IRI+Trib1 NC shRNA group; *,#*p* < 0.05; **,##*p* < 0.01.

To evaluate the role of Trib1 in the adaptive process of repairing moderate AKI *in vivo*, we first established an *in vitro* IRI model using enzymatic method according to previous study (20). The results suggested that IRI model significantly inhibited cell viability of TCMK-1 cells. However, NaSH exposure notably restored the decreased cell viability caused by I/R induction. Of note, Trib1-shRNA mediated the downregulation of Trib1 blunted the recovery effect mediated by NaSH (Supplementary Figure 1). Next, we established a mouse model of moderate AKI to further prove this finding. As shown in Figure 1, compared with those in the sham group, the plasma Scr and BUN levels in the moderate IRI group were increased significantly at day 3 and gradually decreased from day 7 to day 28 after IRI. No significant difference in the levels was observed between the moderate IRI and sham groups from day 7 to day 28 (Figures 2A,B). Interestingly, the levels of Scr and BUN were also increased in the first 3 days and decreased from day 7 to day 28 in the control mice with Trib1 knockdown alone (Figures 2A,B). There was no difference at any time point between the moderate IRI and moderate IRI plus NC shRNA groups. However, compared with those in the moderate IRI and Trib1-knockdown groups, the serum BUN and Scr concentrations were robustly increased in the moderate IRI Trib1-knockdown group on day 3. Although the concentrations of Scr and BUN decreased constantly from day 7 to day 28, they were also significantly greater than those in the moderate IRI group and Trib1-depletion group (Figures 2A,B). Additionally, the concentration of NGAL in the moderate IRI, Trib1-depletion and moderate IRI plus Trib1-depletion groups on day 3 was also significantly higher than that in the sham control group. The NGAL level was also continuously reduced over time. However, unlike the patterns of Scr and BUN, no difference in the NGAL concentration was observed among the moderate IRI, Trib1-depletion and moderate IRI with Trib1-depletion groups on day 3. However, Trib1 inhibition also delayed the decline in the NGAL concentration from day 7 to day 28 during adaptive repair (Figure 2C). These results demonstrate that the inhibition of Trib1 blocks the adaptive repair of renal dysfunction in AKI.

### Inhibition of Trib1 Blunts the Repair of Histological Damage and Fibrosis in Acute Kidney Injury

**Figure 3:**
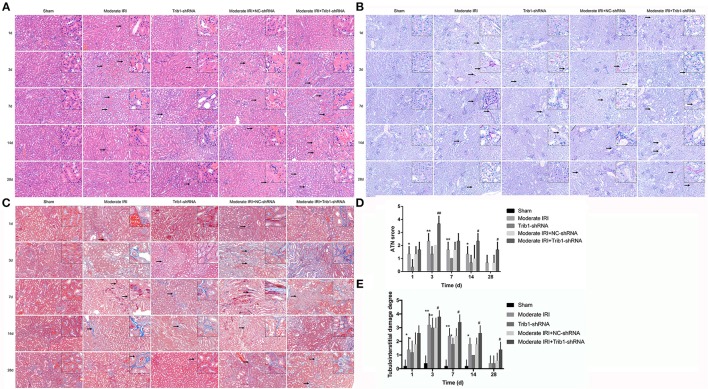
Histopathological detection in mice. **(A)** HE staining of different groups of mice (sham, Trib1 shRNA, moderate IRI, moderate IRI+Trib1 NC shRNA and moderate IRI+Trib1 shRNA groups) at different time points. The arrows indicate the areas of inflammation. **(B)** PAS staining of the five groups of mice. The arrows indicate pathological changes in the renal tissue. **(C)** Masson's trichrome staining of the five groups of mice. The arrows indicate fibrotic areas. **(D)** Acute tubular necrosis (ATN) score in the renal tissue of the above five groups. **(E)** Tubulointerstitial damage degree of the above five groups. Scale bar: 50 μm. * indicated the group of moderate IRI VS the group of Sham. # indicated moderate IRI+Trib1 NC shRNA VS. moderate IRI+Trib1 shRNA. *,#*p* < 0.05; **,##*p* < 0.01.

Next, we assessed renal pathologic changes in moderate AKI mice in the absence of Trib1. The HE and PAS staining results demonstrated that I/R caused significant pathological changes, including severe tubular dilation, tubular cell swelling, TEC shedding, tubular cell necrosis and inflammatory cell infiltration, in moderate IRI mice on day 3. These pathological changes gradually decreased over time from day 7 to day 28 after I/R (Figures 3A,B).

The Trib1-knockdown mice showed the same histopathological variation tendency as the moderate IRI mice. However, compared with IRI mice, the mice with Trib1 depletion subjected to I/R exhibited more serious tubular atrophy and inflammatory cell accumulation on day 3, and numerous necrotic tubular cells and inflammatory cell infiltration remained from day 7 to day 28 (Figures 3A,B). Similarly, I/R-induced renal fibrosis was enhanced on day 3 and eased over time in these mice. However, the Trib1-depleted mice exposed to I/R presented increased collagenous fiber levels and extensive tubulointerstitial damage from day 3 to day 28 when compared with moderate IRI mice and Trib1-knockdown alone mice, implying that Trib1 inhibition impeded the process of repairing histological damage and fibrosis in I/R-induced AKI (Figure 3C). More precisely, we determined overall renal tissue damage according to the ATN score (21). The results indicated that the advanced tissue injury in moderate AKI–mice was observed at days 1 and 3 after I/R, and decreasing gradually from days 7 to 28, which was significantly higher than sham control mice (Figure 3D). Though Trib1 knockdown induced the increase of renal damage at days 1 and 3, the ATN score was notably decreased over time and tended to a more normal rate at day 28 (Figure 3D). However, when moderate IRI mice were exposed to Trib1-shRNA, the robust increase of ATN score was found at days 1 and 3, and Trib1 removal also impeded the process of self-repair in histological damage from days 7 to 28, as compared to moderate IRI mice treated with NC-shRNA (Figure 3D). Next, the damage area including inflammatory cell infiltration area, tubular atrophic area and tubulointerstitial fibrotic area were also used to determine tubulointerstitial damage degree. The tubulointerstitial damage degree was divided into the quality grade 0 to grade 4 based on the damage area. The results indicated that Trib1 depletion or I/R exposure induced the increase of damage area in tubulointerstitial lesions at days 1 and 3, and peaked on day 3. With the increase in time, Trib1 depletion or I/R exposure-mediated the tubulointerstitial damage was recovered based on the self-repair process from day 7 to 28. However, in the absence of Trib1, I/R exposure induced more tubulointerstitial damage from day 1 to 3, and impeded the progression of self-repair from day 7 to 28, showing higher damage degree than I/R exposure alone (Figure 3E).

### Trib1 Depletion Inhibits the Process of Renal Regeneration and Fibrosis Alleviation in AKI Mice

**Figure 4:**
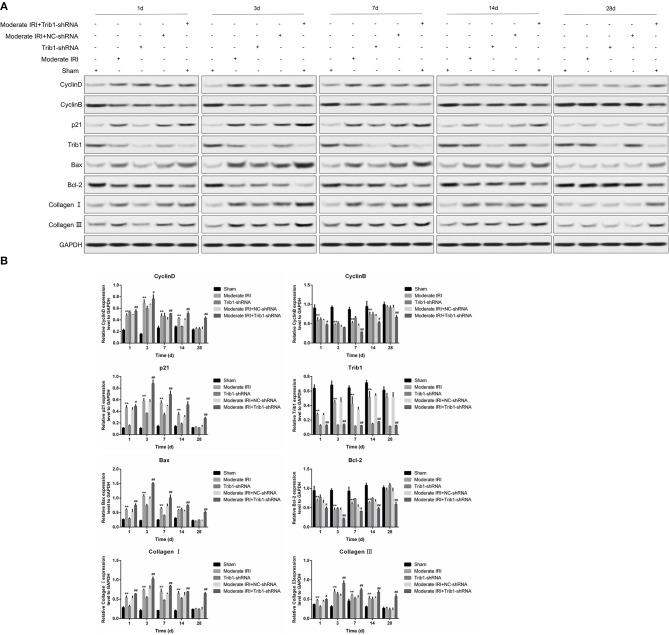
Detection of renal tubular epithelial cell proliferation, apoptosis and fibrosis. **(A)** Protein expression levels of cyclin D1, cyclin B, p21, Bcl-2, Bax, collagen I, collagen III, and Trib1 in the five renal groups were determined by Western blotting at different time points. **(B)** Relative quantitative analysis of the proliferation-related molecules in **(A)**.

Given that renal repair capacity is closely associated with renal interstitial cells loss and proliferation in injured tissue, we measured the expression of proliferation-related molecules in our mouse model. On days 1 and 3 after model establishment, I/R exposure significantly inhibited the expression of Trib1, which was accompanied by a reduction in cyclin B and an increase in cyclin D1 expression (Figure 4A, second and fourth rows vs first row). Trib1 depletion had the same effect as I/R on the expression of Trib1, cyclin D1 and cyclin B on days 1 and 3 (Figure 4A, third row vs. first row). However, decreased cyclin B and increased cyclin D1 expression was observed in Trib1-depleted I/R mice compared to the other four groups of mice (Figure 4A, fifth row vs. other rows). Additionally, the expression of p21, an inhibitor of cyclin-dependent kinases (CDKs), was robustly enhanced in the I/R treatment group; however, its expression slowly increased in the Trib1-knockdown group on days 1 and 3 after model establishment (Figure 4A, second and fourth rows vs. first row). Additionally, the p21 level was highest in the moderate IRI mice treated with Trib1 shRNA on these 2 days (Figure 4A, fifth row vs. other rows). As time progressed, both I/R and Trib1 depletion mediated the decline in Trib1 and cyclin B levels, and the increased cyclin D1 and p21 levels gradually returned to normal on day 28 (Figure 4A). However, the recovery process was prevented by the knockdown of Trib1 in moderate IRI mice, which showed higher cyclin D1 and p21 levels and lower cyclin B levels than mice in the other groups (Figure 4A). In addition, the proapoptotic protein Bax was also induced by I/R and Trib1 knockdown, and its expression was highest in the mice with I/R exposure and Trib1 knockdown on the first 3 days. With the progression of adaptive self-repair, the increase in Bax expression was rescued in both the IRI and Trib1-depleted groups but was also present at a high level in IRI mice treated with Trib1 shRNA. Conversely, the expression pattern of the antiapoptotic protein Bcl-2 was opposite that of Bax under these conditions (Figure 4A). The expression of the mouse renal fibrosis marker collagen I/III was obviously upregulated in the first 3 days and recovered to normal levels on day 28 in the model and Trib1-depletion groups. These changes were blunted by Trib1 depletion in the model group over time (Figure 4A). Using quantitative analyses, we further verified these alterations in proliferation-, apoptosis- and fibrosis-related molecules (Figure 4B). These data reveal that Trib1 is involved in the process of renal tubular regeneration and fibrosis production in AKI.

### Trib1-Knockdown Mice Retain Persistent Inflammation During Renal Adaptive Repair

**Figure 5:**
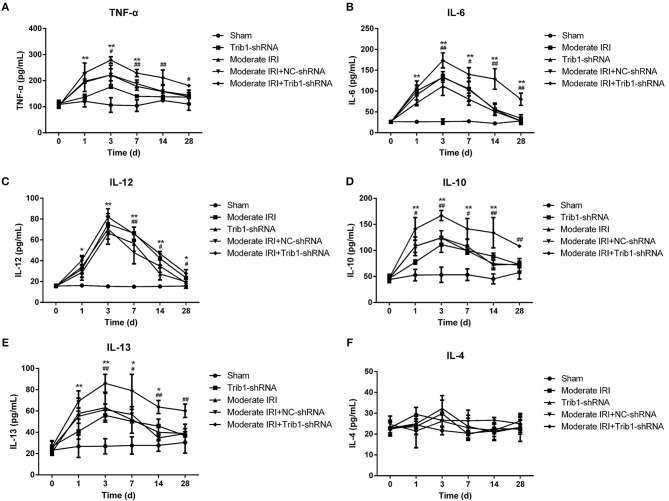
Effect of Trib1 depletion on the secretion of cellular cytokines. Plasma was collected from the above five groups of mice on days 1, 3, 7, 14, and 28 after model establishment. After the serum was obtained, **(A)** TNF-α, **(B)** IL-6, **(C)** IL-12, **(D)** IL-10, IL-13 **(E)**, and IL-4 **(F)** levels were measured by the corresponding ELISA kits. * Indicates the IRI+Trib1 shRNA group vs. the sham group; # indicates the IRI+Trib1 shRNA group vs. the IRI+Trib1 NC shRNA group; *,#*p* < 0.05; **,##*p* < 0.01.

It has been confirmed that an imbalance in the inflammatory microenvironment can completely disrupt the process of renal adaptive repair. Thus, we explored the internal environmental homeostasis of renal tissue in our mouse model. Compared with those in the sham group, the serum levels of TNF-α, IL-6, IL-12, IL-10, and IL-13 were robustly increased in the moderate IRI and Trib1-knockdown groups from day 1 to day 3 and peaked on day 3. The concentrations of these cytokines declined from the peak on day 3 to the normal range, with slightly higher than normal levels on day 28, but no significant difference was found between the cytokines (Figures 5A–E). However, the serum inflammatory factor levels were increased on day 3, and the adaptive self-repair abilities, such as the recovery of serum cytokine levels in IRI mice, were ablated by the absence of Trib1, resulting in increased levels of inflammatory factors in the IRI plus Trib1 shRNA group from day 7 to day 28 (Figures 5A–E). However, no differences in the IL-4 contents in plasma and kidney tissues were observed between the model and Trib1-depletion groups compared to the sham group. The expression of IL-4 in the Trib1 inhibition with IRI group was also not affected compared to that in the other four groups (Figure 5F, Supplementary Figure 2). Therefore, Trib1 inhibition maintains the inflammatory microenvironment after I/R, resulting in the inhibition of renal regeneration.

### Trib1 Is Required for Macrophage Polarization During Renal Adaptive Repair in AKI

**Figure 6:**
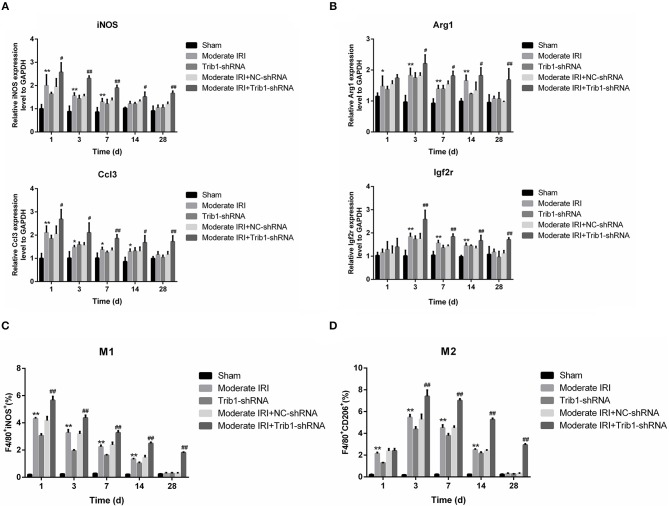
Effect of Trib1 inhibition on macrophage polarization in mice. **(A)** The relative expression of iNOS and Ccl3 was detected by qRT-PCR. **(B)** The relative expression of iNOS and Ccl3 was analyzed by qRT-PCR. **(C)** Statistical analysis of the proportion of cells labeled with F4/80^+^iNOS^+^ in the five groups. **(D)** Statistical analysis of the population of cells labeled with F4/80^+^CD206^+^ in the five groups; *,#*p* < 0.05; **,##*p* < 0.01.

Macrophage polarization is a critical step in the regulation of AKI inflammation. The ratio of M1 to M2 macrophages determines the internal environmental homeostasis of renal tissue, and the secretion of cellular inflammatory cytokines contributes to the polarization of macrophages. As shown in Figure 5, the expression of M1 macrophage markers, iNOS and Ccl3, was robustly increased in the IRI and IRI+Trib1 shRNA groups on day 1 and was highest in the IRI+Trib1 shRNA group compared to the sham group. However, over time, the levels of both iNOS and Ccl3 decreased to normal in the IRI and Trib1 shRNA groups, whereas the expression levels of these markers remained high in IRI mice treated with Trib1 shRNA (Figure 6A). However, the expression levels of the M2 macrophage markers Arg1 and Igf2r were notably enhanced in the IRI and IRI+Trib1 shRNA groups on day 3, and the expression of only Arg1 was significantly increased on day 1. Both Arg1 and Igf2r were highly expressed in the IRI+Trib1 shRNA group compared to the IRI and sham groups (Figure 6B). Consistently, Arg1 and Igf2r expression decreased over time and recovered to normal baseline levels on day 28 after establishment of the model. Conversely, the inhibition of Trib1 also blocked the IRI adaptive repair process from day 7 to day 28 (Figure 6B). Furthermore, the number of F4/80^+^iNOS^+^-positive cells (M1 macrophages) increased significantly after IRI and Trib1 depletion on the first day and approached normal levels over time. However, Trib1 knockdown promoted an increase in F4/80^+^iNOS^+^-positive cell numbers on the first day and impeded the decline in numbers from day 3 to day 28 (Figure 6C, Supplementary Figure 3). In contrast, the increase in the number of F4/80^+^CD206^+^-positive cells (M2 macrophages) appeared in the first 3 days and subsequently fell slowly from day 7 to day 28 in the IRI group, but these changes were blocked by Trib1 depletion (Figure 6D, Supplementary Figure 4). These data show that Trib1 is involved in the process of macrophage polarization during renal adaptive repair in AKI.

## Discussion

In the present study, we verified that the inhibition of Trib1 resulted in markedly reduced cell viability in renal tubular epithelial cells; and renal dysfunction and severe renal pathological damage in IRI kidneys. Furthermore, Trib1 removal also blunted the regeneration of renal tubular and perturbs internal environmental macrophage polarization homeostasis during repair after I/R-mediated injury. Our findings clarify the role of Trib1 in the recovery of I/R-induced AKI and its link with macrophages in this regulation. It is possible that targeting Trib1 would facilitate the treatment of AKI.

The mammalian Tribbles (Trib) family contains Trib1, Trib2, and Trib3, which participate in the degradation of target proteins and the regulation of signal transduction (22). As a mammalian ortholog of Tribbles, Trib1 controls cell division and migration during embryonic development in Drosophila (23, 24). Increasing evidence suggests that the loss of Trib1 is conducive to injury repair and tissue remodeling. It has been proven that Trib1 plays an important regulatory role in vascular smooth muscle cell proliferation via the MAPK activation-mediated Jun kinase pathway (25). The loss of Trib1 also represses liver fibrosis repair (16). Thus, Trib1 might act as a key switch for injury repair and cell regeneration. In the present study, Trib1 depletion depressed cell viability by inhibiting proliferation of renal tubular epithelial cells and inducing cell apoptosis *in vitro*, caused the same symptoms of renal dysfunction as I/R administration *in vivo*, and Trib1 depletion or I/R stimulation-induced renal dysfunction was restored by the self-repair systems in mice. Interestingly, Trib1 inhibition obviously counteracted the self-repair process in IRI mice. These observations reveal that Trib1 is a potential antagonist of the complete repair process after AKI, and forced Trib1 expression might accelerate the renal regeneration process. Actually, though Trib1 knockdown blunted cell viability of TECs by inhibiting proliferation and enhancing apoptosis, the inhibition of Trib1 could also cause all renal tubular injury *in vivo*. Thus, knockout of the Trib1 in the renal tubular epithelial cells in mice needed to be established in the future to better demonstrate the correlation with Trib1 and TECs during the progression of IRI *in vivo*.

A previous study demonstrated that overexpression of Tribbles in imaginal disc cells blocks the cell cycle at the G2 phase, resulting in abnormal wing morphology (24). Tribbles also act as a mitotic inhibitor that impedes string at the posttranslational level (26). The interaction of Trib1 with MEK1 enhances the phosphorylation of ERK1/2, leading to the promotion of cell proliferation and the inhibition of apoptosis. Trib1 is a novel regulator of the G1-S transition that regulates the cyclin D1 promoter via NF-κB and AP-1 sites (27). Herein, Trib1 depletion inhibited the expression of cyclin B and increased the level of p21 to the same extent as I/R treatment, whereas Trib1 depletion and I/R upregulated cyclin D1 expression in the first 3 days of AKI. Although these changes were rescued over time, the knockdown of Trib1 delayed the self-repair process. Cyclin B, as a regulator of the G2/M phase, is expressed from the S phase; its expression peaks at the G2/M phase and disappears in other cell cycle phases (28). p21 is associated with G1/S and G2/M phase transitions through binding to CDKs. An increase in p21 expression occurs on the first day in 2w-AKI mice but not in long-term AKI mice (29). However, cyclin D1 is a mid-late G1 phase marker. Enhanced G1 arrest is associated with the modulation of cell cycle-regulating factors, including cyclin D1, p21, and p27, in AKI (30). Thus, we hypothesized that Trib1 depletion and I/R would induce cell cycle arrest in the G1 phase, decrease the G2/M and S phase cell populations, increase Cyclin D1 and p21 expression and decrease cyclin B expression.

The inflammatory response is involved in IRI and the renal repair process. In response to prolonged ischemia followed by reperfusion, TECs secrete many proinflammatory cytokines, such as IL-6 and tumor necrosis factor (TNF)-α, and leucocytes, such as neutrophils, dendritic cells, natural killer (NK) cells, T cells and macrophages, infiltrate the injury sites (31). The accumulated macrophages are responsible for producing proinflammatory cytokines, including IL-6 and TNF-α, resulting in inflammation and tissue damage (M1 macrophages), and for clearing cellular debris and apoptotic and necrotic cells. This clearance eliminates the excessive deposition of extracellular matrix via the secretion of anti-inflammatory cytokines, such as IL-4, IL-10 and IL-13, thus leading to the promotion of normal structure and restoration of function in the injured tissue (M2 macrophages) (11, 32). In I/R-induced AKI, an early increase in renal inflammatory (M1) monocytes occurs, followed by an aggregation of renal macrophages with a wound-healing (M2) phenotype (33). Notably, Trib1 plays an important role in the regulation of the inflammatory response. Trib1 is a critical factor for adipose tissue maintenance and the suppression of metabolic disorders that act by controlling the differentiation of tissue-resident M2-like macrophages (34). However, whether Trib1 is involved in the inflammatory response in I/R-mediated AKI remains unclear. In this study, along with the development of I/R and Trib1 knockdown-mediated renal injury, the expression levels of proinflammatory cytokines, including TNF-α, IL-6, and IL-12, and the anti-inflammatory cytokines IL-10 and IL-13 were increased from day 1 to day 3 and gradually decreased over time. This cytokine expression increase was accompanied by an increase in M1 macrophages on day 1 and in M2 macrophages from day 1 to day 3, implying that M2 accumulation occurs later than M1 accumulation and that the activity of the M2 phenotype macrophages remains for a prolonged time period. M2 macrophages control the renal repair process after IRI, a phenomenon that further explains the self-adaptation mechanism of renal AKI. Although a decrease in M2 macrophage numbers was observed after model establishment over time, the ratio of M1/M2 macrophages was also low, suggesting that M2-mediated anti-inflammatory effects play a primary role in the repair process. Without affecting IL-10 and IL-13, the administration of IL-4 is known to increase the number of M2 macrophages (35). Alternatively, activated M2 macrophages induced by IL-4 stimulation are involved in cardiac repair after myocardial infarction (36). Here, we did not observe changes in serum IL-4 levels in the different groups of mice regardless of IRI or Trib1 depletion. Therefore, the mechanism underlying adaptive self-repair induced by M2 macrophage activation may be associated with the increase in IL-10, but not IL-4, expression in the moderate AKI model. Additionally, we hypothesized that the inhibitory role of Trib1 in the process of self-repair also occurs through the regulation of the M1/M2 macrophage ratio because this ratio was higher in the IRI+Trib1_siRNA group than in the sham, IRI, Trib1 shRNA and IRI+Trib1 shRNA NC groups and was accompanied by a decline in M1 and M2 macrophages during the self-repair process. Collectively, these results indicate that Trib1 mediates the self-repair of AKI by regulating macrophage polarization.

## Conclusion

Our findings demonstrate that Trib1 inhibition promotes renal injury by regulating the regeneration of renal tubular, the secretion of inflammatory cytokines and macrophage polarization. Moreover, Trib1 depletion blocks the renal repair process after IRI by inhibiting epithelial cell growth and promoting apoptosis and fibrosis by repressing the ratio of M1/M2 macrophages. It is possible that targeting Trib1 may offer a new treatment approach for AKI.

## Data Availability Statement

All datasets generated for this study are included in the article/Supplementary Material.

## Ethics Statement

The animal study was reviewed and approved by Ethics Committee of Hangzhou First People's Hospital.

## Author Contributions

XF and XY conducted the research. JW and XX drafted the paper. WW and JM analyzed the data and performed the statistical analysis. XX and MW designed the research protocols.

### Conflict of Interest

The authors declare that the research was conducted in the absence of any commercial or financial relationships that could be construed as a potential conflict of interest.

## Supplementary Material

Supplementary Figure 1 Effect of Trib1 knockdown on cell viability in *in vitro* IRI model using renal tubular epithelial cells. Cells were pre-treated with 1 mM NaSH for 1 h before the induction of 1 h ischemia and 3 h reperfusion. Cell viability was determined by CCK8 assay. I/R: 1 h ischemia and 3 h reperfusion; NaSH: positive control and NaCl: negative control. ^*^*p* < 0.05; ^**^*p* < 0.01.

Supplementary Figure 2 Relative expression of IL-4 in kidney tissue of different group. The mice were randomly divided into five groups: sham, Trib1 shRNA, moderate IRI, moderate IRI+Trib1 NC shRNA and moderate IRI+Trib1 shRNA. Kidney tissues were collected at each time points after I/R (days 1, 3, 7, 14, and 28).

Supplementary Figure 3 The regulatory effect of Trib1 on the number of M1 macrophages. The number of M1 macrophages labeled with F4/80^+^iNOS^+^ in the five groups on days 1, 3, 7, 14, and 28 was determined by flow cytometry.

Supplementary Figure 4 The regulatory effect of Trib1 on the number of M2 macrophages. The number of M2 macrophages labeled with F4/80^+^CD206^+^ in the five groups at different time points was detected by flow cytometry.

## Abbreviations

AKI: acute kidney injury
I/R: ischemia-reperfusion
IRI: ischemia-reperfusion injury
RNAi: RNA interference
CKD: chronic kidney disease
ESRD: end-stage renal disease
TEC: tubular epithelial cell
Trib1: Tribbles homolog 1
GFP: green fluorescent protein
Scr: creatinine
BUN: blood urea nitrogen
NGAL: neutrophil gelatinase-associated lipocalin
HE: hematoxylin-eosin
PAS: periodic acid-Schiff
SDS-PAGE: sodium dodecyl sulfate-polyacrylamide gel electrophoresis
PVDF: polyvinylidene difluoride membranes
TNF-α: tumor necrosis factor
NK: natural killer cells
ATN: acute tubular necrosis.
