# Supplementary figures and images for: Trib1 Contributes to Recovery From Ischemia/Reperfusion-Induced Acute Kidney Injury by Regulating the Polarization of Renal Macrophages
Source: Front Immunol. 2020 Mar 20;11:473. doi: 10.3389/fimmu.2020.00473 (PMC7098949; doi:10.3389/fimmu.2020.00473)

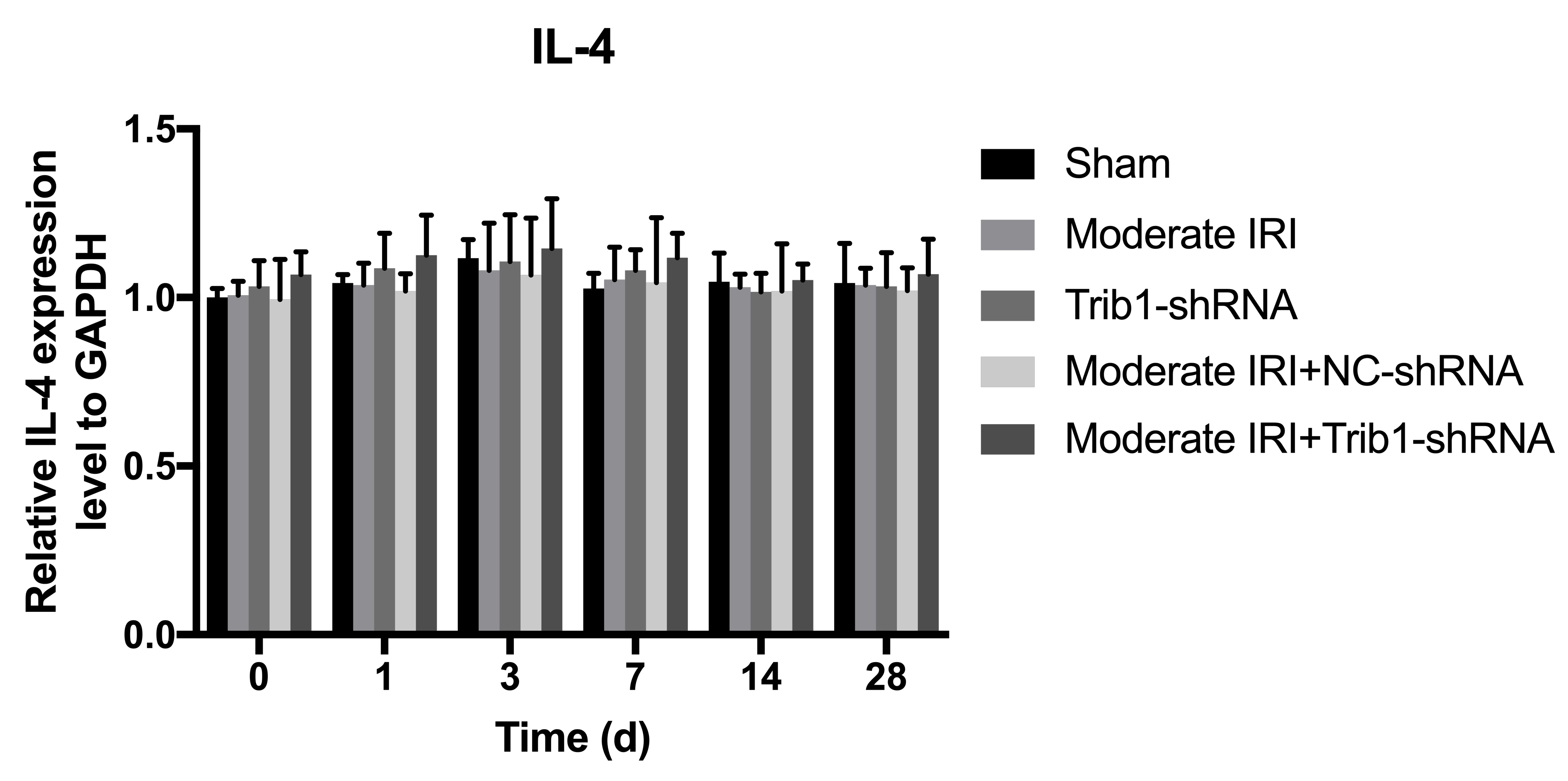

Supplement: Supplementary Figure 2 — Relative expression of IL-4 in kidney tissue of different group. The mice were randomly divided into five groups: sham, Trib1 shRNA, moderate IRI, moderate IRI+Trib1 NC shRNA and moderate IRI+Trib1 shRNA. Kidney tissues were collected at each time points after I/R (days 1, 3, 7, 14, and 28). [file Image_2.TIFF]

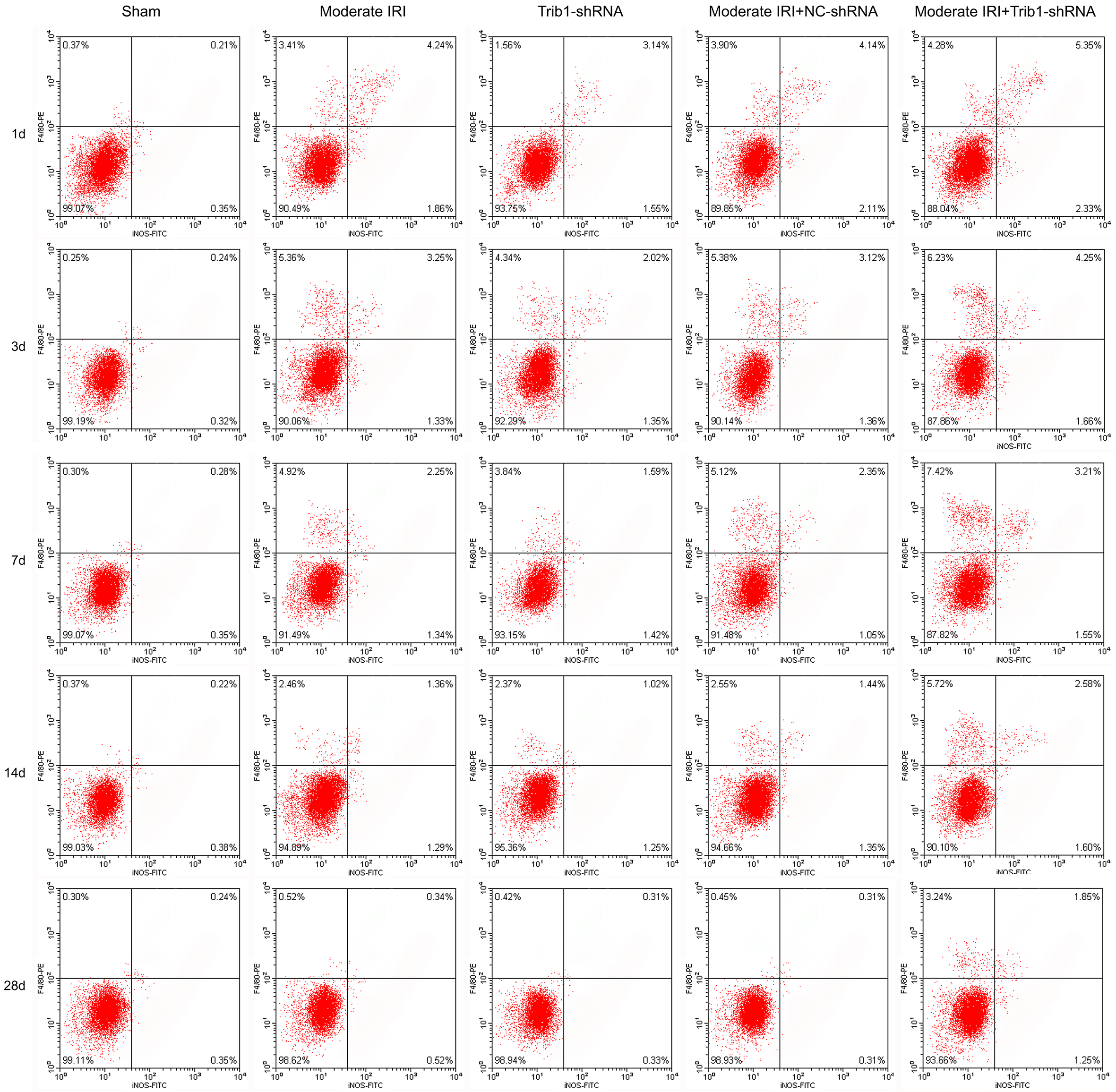

Supplement: Supplementary Figure 3 — The regulatory effect of Trib1 on the number of M1 macrophages. The number of M1 macrophages labeled with F4/80+iNOS+ in the five groups on days 1, 3, 7, 14, and 28 was determined by flow cytometry. [file Image_3.TIF]

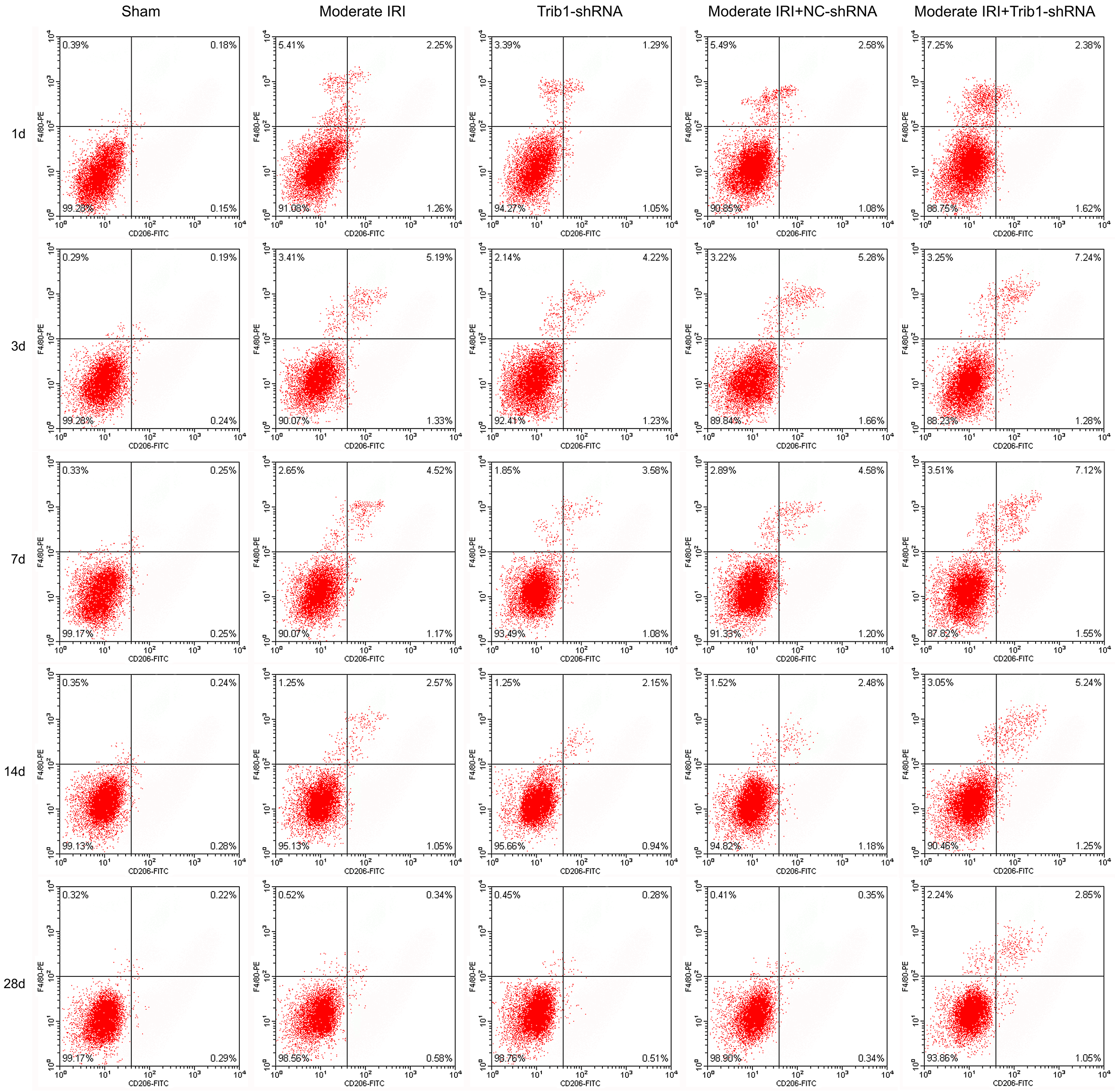

Supplement: Supplementary Figure 4 — The regulatory effect of Trib1 on the number of M2 macrophages. The number of M2 macrophages labeled with F4/80+CD206+ in the five groups at different time points was detected by flow cytometry. [file Image_4.TIF]
